# Recent progress on magnetic nanoparticles for magnetic hyperthermia

**DOI:** 10.1007/s40204-016-0054-6

**Published:** 2016-09-06

**Authors:** Lina Kafrouni, Oumarou Savadogo

**Affiliations:** 1 Department of Chemical Engineering, Polytechnique Montréal, C.P. 6079, Succursale Centre-ville, Montreal, QC H3C 3A7 Canada; 2 Laboratory of New Materials for Energy and Electrochemistry Systems (LaNoMat), Montreal, Canada

**Keywords:** Magnetic nanoparticles, Synthesis, Magnetic hyperthermia, Cancer

## Abstract

Recent advances in nanomaterials science contributed to develop new micro- and nano-devices as potential diagnostic and therapeutic tools in the field of oncology. The synthesis of superparamagnetic nanoparticles (SPMNPs) has been intensively studied, and the use of these particles in magnetic hyperthermia therapy has demonstrated successes in treatment of cancer. However, some physical limitations have been found to impact the heating efficiency required to kill cancer cells. Moreover, the bio-safety of NPs remains largely unexplored. The primary goals of this review are to summarize the recent progress in the development of magnetic nanoparticles (MNPs) for hyperthermia, and discuss the limitations and advances in the synthesis of these particles. Based on this knowledge, new perspectives on development of new biocompatible and biofunctional nanomaterials for magnetic hyperthermia are discussed.

## Introduction

According to the National Cancer Institute, cancer is currently the second leading cause of death in the United States, exceeded only by heart disease as the number one killer. A total of 1,620 Americans are expected to die of cancer per day in 2015.

Significant progress has been made so far in nanotechnology for the diagnosis and treatment of cancer. A variety of magnetic nanomaterials has been developed to achieve improved efficacy in cancer therapy as well as reduced side effects compared to conventional therapies. The interest in MNPs is due to their unique magnetic properties; they exhibit diagnostic tool, drug carrier and heat generator for therapy in magnetic resonance imaging (MRI), so-called ‘theranostic’ and their small sizes, which allow the particles to reach most biological tissues. Currently, iron oxide nanoparticles (IONPs) are the most explored MNPs for magnetic hyperthermia, because of their lack of toxicity and their known pathways of metabolism (Tran et al. 2012a, b).

The generation of heat by the exposition of MNPs to a non-invasive alternating magnetic field (AMF) can be used to destroy tumor tissue, given that heat promotes cell apoptosis through irreversible physiological changes (Prasad et al. 2007). This approach is known as magnetic hyperthermia. The basics of the magnetic properties required in MNPs for magnetic hyperthermia applications will be discussed later in detail.

The synthesis methods of MNPs have an impressive impact on the magnetic and morphological properties of the final product (Castellanos-Rubio et al. 2015). Therefore, a synthesis method with the ability to rigorously control the composition, size and shape is needed. This paper presents a short review on the current methods for synthesis of MNPs for nanomedicine, and discusses important findings reported earlier.

## Basics of magnetism in magnetic hyperthermia

An understanding of the relationship between physicochemical properties (for example: structure, particle size) and magnetic properties is essential to design new magnetic materials for magnetic hyperthermia applications. Therefore, a review on the basic concepts in nano-magnetism will be discussed shortly.

### Soft and hard magnets

When a ferromagnetic material, such as Iron, nickel and cobalt, is placed in a magnetic field of strength ‘H’, the atoms acquire an induced magnetic moment ‘m’ randomly oriented. The magnetic moments pointed in the same direction per volume of atoms are called magnetization ‘M’. The magnetic induction ‘B’ is given by Maxwell’s equation (Eq. 1) (Laurent et al. 2011).

1 $$ B = \mu_{0} \left( {H + M} \right) $$

where $\mu_{0}$ is the permeability of the free space which equals to 4π $10^{ - 7}$ V.s/A.m.

**Fig. 1:**
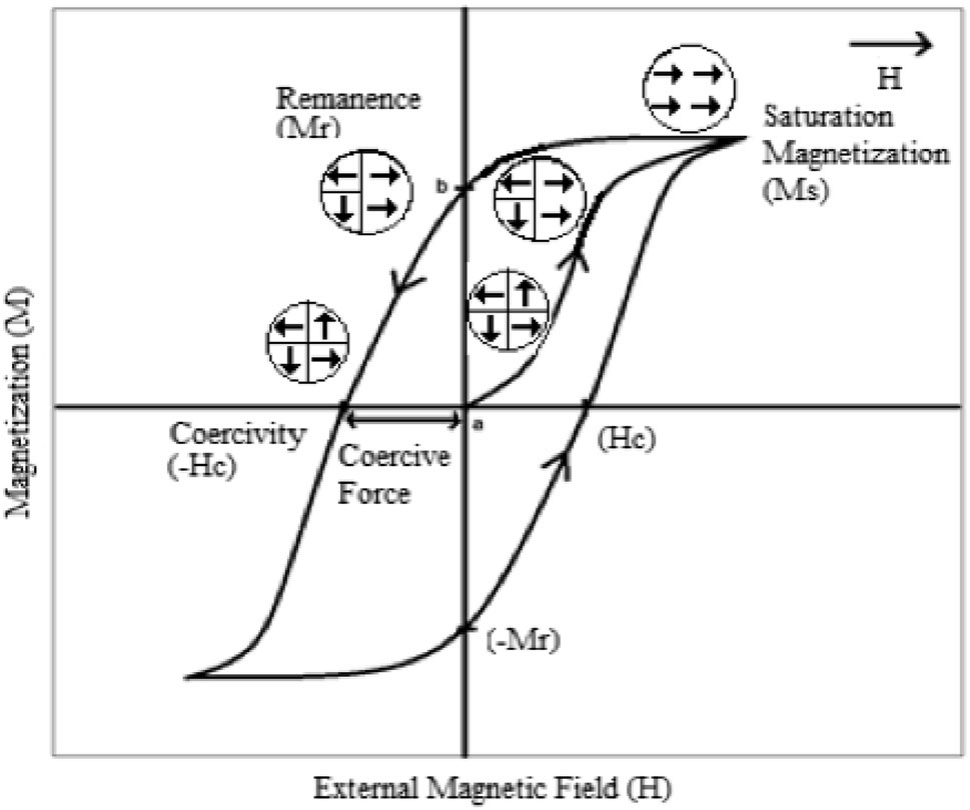
Typical hysteresis loop of ferromagnetic materials (adapted from Mody et al. 2013)

The small regions of magnetization are called magnetic domains, and the boundaries between domains are called domain walls. In the absence of an external magnetic field, ferromagnetic material does not show any magnetization due to the random orientation of the magnetizations in magnetic domains (Point a, Fig. 1). However, when an external magnetic field is applied, magnetic moments become aligned to the direction of the magnetic field, so the domain walls disappear and the magnetization becomes saturated, the so-called saturation magnetization (Ms) (Fig. 1).

Once the applied magnetic field is removed, ferromagnetic materials keep some memory of the applied field (Point b, Fig. 1), called remanence (Mr). A coercive force must be applied to reduce the remanent magnetization to zero and close the loop.

Ferromagnetic materials can be categorized into soft and hard magnets (Mody et al. 2013). Soft magnets have a low coercivity (Hc), so they can be demagnetized at low magnetic field. However, hard magnets exhibit a high Hc and thus they are difficult to demagnetize.

### Multi-domain to single domain

**Fig. 2:**
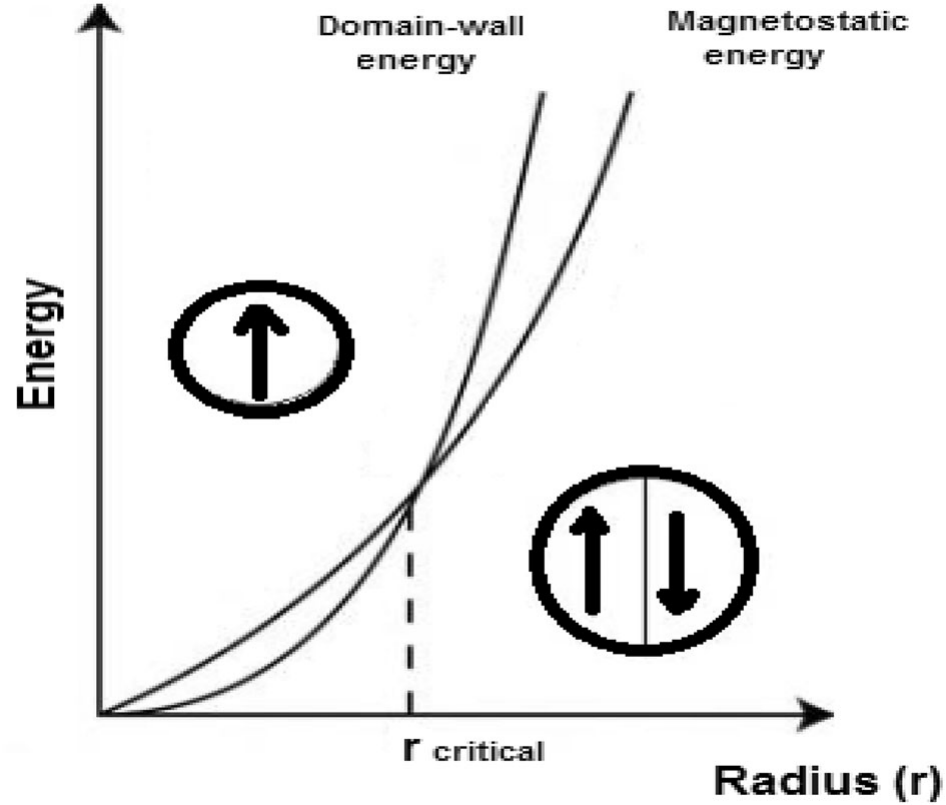
Relative stability of multi-domain and single domain (adapted from Spaldin 2011)

The magnetostatic (dipole–dipole) energy is inversely proportional to the volume of the particle ($r^{3}$), and the domain-wall energy is proportional to the area of the wall ($r^{2}$) (Fig. 2) (Spaldin 2011).

By looking at the balance between the magnetostatic energy and the domain wall energy, it is energetically unfavorable to form domain walls below a critical radius, because the domain-wall energy is very low, and a single domain is formed as a result of high magnetostatic energy.

For a sphere containing two semi-sphere domains of opposite magnetization with axial magnetic anisotropy, the critical single-domain radius is given by Eq. (2) (Skomski 2003).

2 $$ r_{\text{critical}} = \frac{{36\sqrt {AK_{1} } }}{{\mu_{0} M_{s}^{2} }} $$

where *A* is the exchange stiffness and $K_{1}$ is the first uniaxial anisotropy constant.

The critical radius values corresponding to ferromagnetic elements Fe, Co and Ni are calculated according to Eq. (2) and are presented in Table 1.

**Table 1:** Magnetic parameters at room temperature (Skomski 2003)

Ferromagnetic particles	Fe	Co	Ni
A (pJ/m)	8.3	10.3	3.4
K1 (MJ/${\text{m}}^{3}$)	0.05	0.53	−0.005
μ_0_·M_s_ (T)	2.15	1.76	0.61
*r*_C _(nm)	6	34	16

### Superparamagnetism

**Fig. 3:**
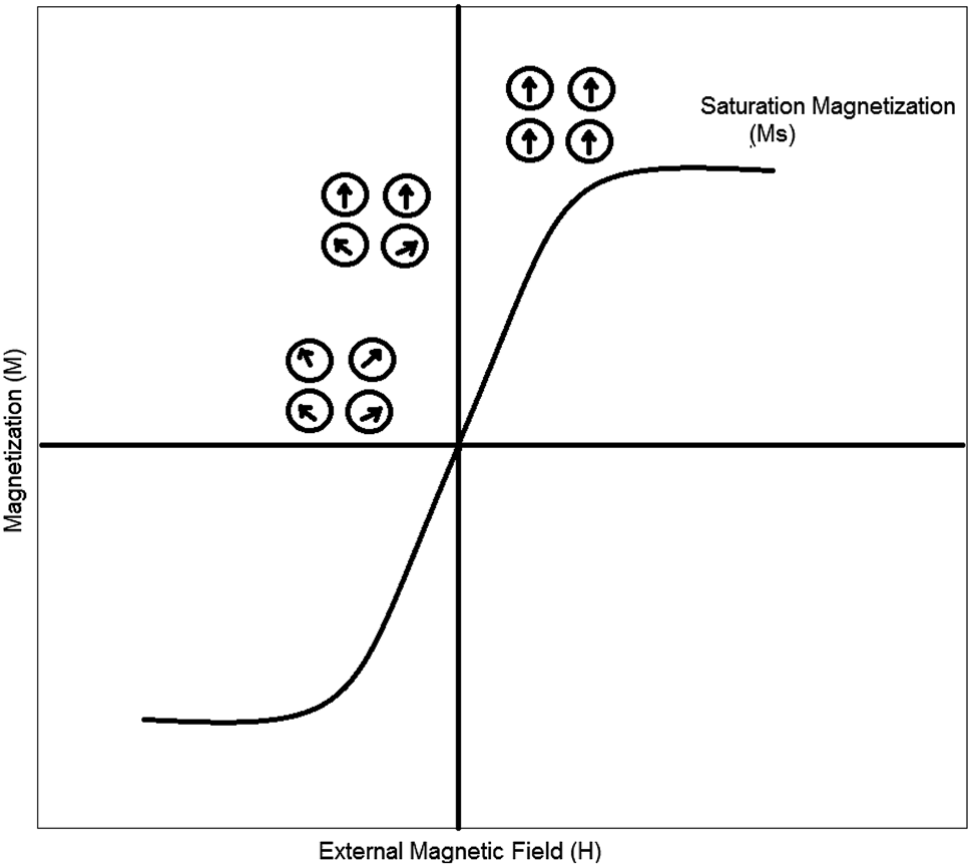
The magnetic response characteristic of a superparamagnetic material (adapted from Mody et al. 2013)

It has been found that with a further decrease in particle size below the critical radius, the coercivity Hc decreases significantly to reach zero. When the coercivity becomes zero, the particles magnetize in the presence of an external magnetic field and revert to a non-magnetic state when the external magnetic field is removed (Fig. 3) (Mody et al. 2013).

This behavior can be explained by the fact that a small magnetic particle less than critical size prefers to be uniformly magnetized along one of its easy axes ($\theta$ = 0, $\theta$ = *π*), and the energy required to rotate the magnetization away from the easy direction is called magnetic anisotropy energy. In a simple model for a non-interacting single-domain spherical particle with uniaxial anisotropy in zero magnetic field, the magnetic anisotropy energy ‘$E_{A}$’ is given by an expression of Eq. (3) (Stoner and Wohlfarth 1948).

3 $$ E_{A} = {\text{K}} \cdot {\text{V}} \cdot \sin^{2} \theta $$

where *K* is the anisotropy constant, *V* is the volume of the particle and *θ* is the angle between the particle magnetization and the easy magnetization axis of the particle.

**Fig. 4:**
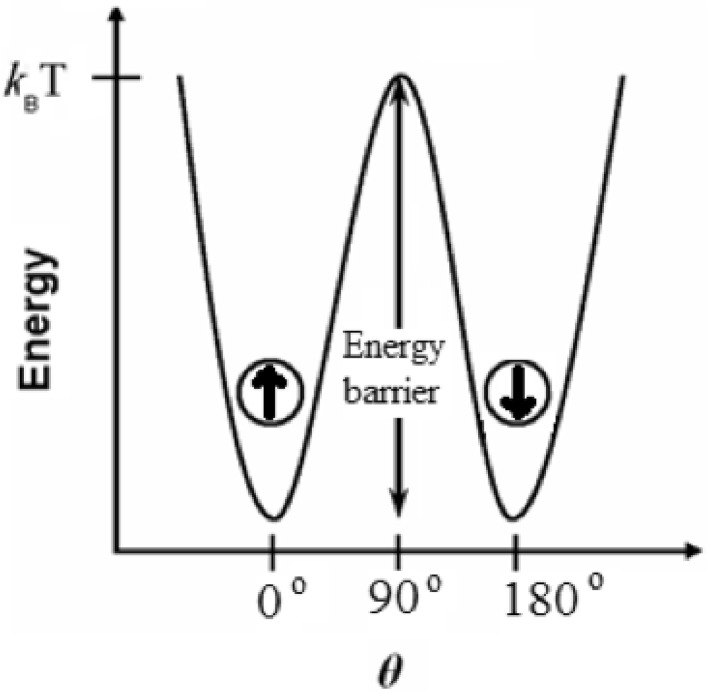
Schematic of anisotropy energy barrier for magnetization reversal (adapted from Stoner and Wohlfarth 1948)

According to Eq. (3), the magnetic anisotropy energy decreases when the volume of the particle becomes smaller. Furthermore, the anisotropy energy becomes comparable to or even lower than the thermal energy ($E_{\text{thermal}}$ = $k_{B}$·*T*, where $k_{B}$ is Boltzmann constant) (Krishnan 2010). As a result, the energy barrier for magnetization reversal can be overcome thermally (Fig. 4). This phenomenon is called ‘superparamagnetism’.

Due to the fact that these particles are magnetically controlled by an external magnetic field and maintain a colloidal stability upon removal of the external magnetic field, superparamagnetic particles have a unique advantage for biomedical applications.

For spherical magnetic particles, the transition from single domain to superparamagnetic ‘$r_{0}$’ depends on the size and/or geometry of the particles and can be determined by the following Eq. (4) (Martel 2015):

4 $$ r_{0} = \left( {\frac{{6 \cdot k_{B} \cdot T_{B} }}{K}} \right)^{1/3} $$

where $T_{B}$ is the blocking temperature.

Table 2 provides calculated values of the transition radius ‘$r_{0}$’, according to Eq. (4), for the main magnetic nanomaterials (Martel 2015; Kolhatkar et al. 2013).

**Table 2:** Maximum radius for superparamagnetic NPs of different compositions (Martel 2015; Kolhatkar et al. 2013)

Superparamagnetic NPs	Co	CoPt	Co ${\text{Fe}}_{2} {\text{O}}_{4}$	FeCo	${\text{Fe}}_{3} {\text{O}}_{4}$	${\text{Fe}}_{2} {\text{O}}_{3}$	FePt	Ni
*r*_0_ (nm)	5	1	5	10	12.5	15	1.5	15

Although particle moves toward superparamagnetism when the size of the particle decreases below the transition point and becomes suitable for biomedical application, the saturation magnetization ‘Ms’ reduces. The magnitude of Ms is inversely proportional to the ratio of disordered spin layer at the surface to the radius of the particle, which significantly increases when the size of the nanoparticle becomes too small. The relationship between Ms, the size and the disordered spin layer is described by Eq. (5) (Jun et al. 2008):

5 $$ {\text{Ms }} = {\text{Ms}}_{\text{b}} \left[ {\frac{(r - d)}{r}} \right]^{3} $$

where *d* is the thickness of the particle’s surface exhibiting disordered spins, and ${\text{Ms}}_{\text{b}}$ is the bulk Ms. Recent studies on the effect of the size of MNPs upon its saturation magnetization are summarized in Table 3. According to the studies listed in Table 3, the Ms increases with the size of the MNPs due to the reduction of the spin disorder effect.

**Table 3:** Magnetizations of a variety of types of MNPs of varying sizes

MNPs	Size (nm)	Ms (emu/g)	References
Co ${\text{Fe}}_{2} {\text{O}}_{4}$	4.2	30.6	Pereira et al. (2012)
	4.8	46.0	
	18.6	48.8	
${\text{Fe}}_{3} {\text{O}}_{4}$	4.9	60.4	
	6.3	64.8	
Ni	24	25.3	He and Shi (2012)
	50	32.3	

Recent study done by Guardia et al. have demonstrated that the surface coating of iron oxide (${\text{Fe}}_{3} {\text{O}}_{4}$) NPs with oleic acid increases their measured Ms to reach the bulk value, by reducing the level of surface spin disorder (Guardia et al. 2007).

### Heat generation

Heating tumor cells with SPMNPs by magnetic hyperthermia is based on Neel and Brownian relaxations. In the presence of an external alternating magnetic field, the magnetic moment rotates and the nanoparticle itself rotates, then relaxes back to their original magnetic field orientation. The rotation of the magnetic moment (Neel mode) and the friction arising from particle oscillations (Brownian mode) leads to a phase lag between applied magnetic field and the direction of the magnetic moments. As a result, the heat is released.

The efficiency of heating is measured in terms of the specific absorption rate (SAR), or specific loss of power (SLP), which is defined in Eq. (6). For biomedical applications, the value of SAR is crucial, because the higher the specific absorption rate, the lower the injected dose to the patient.

6 $$ {\text{SAR or SLP }}\left( {{\text{W}}/{\text{g}}} \right) \, = C\frac{\Delta T}{\Delta t} $$

where *C* is the specific heat capacity of water, and Δ*T*/Δ*t* is the rate of change of temperature versus time.

According to Rosensweig (2002), there is a strong relationship between the SAR of SPMNPs and its magnetic relaxation ‘$\tau$’ (Eq. 7).

7 $$ {\text{SAR}} = 4.1868\pi \mu_{0}^{2} \frac{{\varphi M_{s}^{2} V}}{1000kT} \cdot H_{0}^{2} v\frac{2\pi \nu \tau }{{1 + (2\pi \nu \tau )^{2} }} $$

where *φ* is the volume fraction of the SPMNPs, *V* = $\frac{{4\pi r^{3} }}{3}$ is the magnetic volume for a particle of radius *r*, $H_{0}$ is the magnetic field intensity, *ν* is the frequency of the oscillating magnetic field and *τ* is the relaxation time. The other parameters (*μ*_o_ is the permeability of the free space), *π*, *k* (Boltzmann constant) and *T* (temperature of the sample) have their classical meanings.

Also, Eq. (7) shows that the SAR strongly depends on the $M_{s}$ and the volume fraction of the SPMNPs. Not only high $M_{s}$ values are required for thermal energy dissipation in the tumor cells, but also to give more control on the magnetophoretic velocity of the MNPs ‘$V_{\text{mag}}$’ in the blood using external magnetic field (Grief and Richardson 2005) (Eq. 8).

8 $$ V_{\text{mag}} = \frac{{M_{s} \cdot V_{\text{microdevice}} \cdot \nabla B}}{{6 \cdot \pi \cdot R_{\text{microdevice}} \cdot \mu }} $$

where $V_{\text{microdevice}}$ is the volume of microdevice (${\text{m}}^{3}$), $\nabla B$ is the magnetic gradient applied (*T*/*m*), $R_{\text{microdevice}}$ is the microdevice radius (*m*) and $\mu$ is the blood viscosity (Pa.s).

Theoretically, a critical diameter $d_{c}$ is defined as the diameter for which the Neel relaxation time ‘$\tau_{N}$’ (Eq. 9) is equal to the Brownian relaxation time ‘$\tau_{B}$’ (Shliomis and Stepanov 1990) (Eq. 10). For small particles with a diameter <$d_{c}$, Neel relaxation is predominant. However, the heating is primarily due to Brownian rotation in larger particles with a diameter >$d_{c}$. The dominating contribution will be by the faster relaxation time.

9 $$ \tau_{N} = \tau_{0} e^{{\frac{K \cdot V}{{k_{B} \cdot T}}}} $$

10 $$ \tau_{B} = \frac{{3 \eta V_{B} }}{{k_{B} \cdot T}} $$

where *K* is the anisotropy constant of magnetite which is over the range of 23,000–100,000 J m^−3^, while $\tau_{0}$ ≈ $10^{ - 9}$–$10^{ - 12}$ s is the relaxation time of non-interacting MNPs, *η* is the viscosity of the surrounding liquid and $V_{B}$ is the hydrodynamic volume of the particle; *k*_*B*_ is the Boltzmann constant and *T* is the temperature of the sample.

The frequency $\nu_{N}$ for maximal heating via Neel relaxation is given by Eq. (11), and the frequency $\nu_{B}$ for maximal heating via Brown rotation is given by Eq. (12) (Fannin and Charles 1991).

11 $$ 2\pi \nu_{N} \tau_{N} = 1 $$

12 $$ 2\pi \nu_{B} \tau_{B} = 1 $$

When the diameter of the particle is close to $d_{c}$, $\tau_{N}$ ≈ $\tau_{B}$ and an effective relaxation time ‘$\tau_{\text{eff}}$’ is defined in Eq. (13). The frequency for maximal heating ‘$\nu_{\text{eff}}$’ is then given by Eq. (14) (Fannin et al. 1993).

13 $$ \tau_{\text{eff}} = \frac{{\tau_{N} \tau_{B} }}{{(\tau_{N} + \tau_{B} )}} $$

14 $$ 2\pi \nu_{\text{eff}} \tau_{\text{eff}} = 1 $$

Recent research optimized the heating efficiency by tuning the MNPs size to match the total relaxation time ($\tau_{\text{total}}$ = $\tau_{\text{N}}$ + $\tau_{B}$) to the applied frequency (ν = $\frac{1}{{2\pi \cdot \tau_{\text{total}} }}$) (Khandhar et al. 2011).

The strong dependence of the SAR on multiple magnetic properties such as saturation magnetization and relaxation time, physical parameters like size, shape and composition can be tailored to enhance the heat dissipation and thus lower the injected dose of SPMNPs in the tumor site.

## Biomaterials for magnetic hyperthermia

To develop excellent candidates for magnetic hyperthermia, it is very important to review the recent advances and limitations in the development of MNPs for magnetic hyperthermia applications.

Superparamagnetic iron oxide nanoparticles (SPIONs) are the most used MNPs for biomedical applications, especially magnetic hyperthermia. They received considerable attention due to their biocompatibility compared to other magnetic materials such as cobalt and nickel (Tran et al. 2012a, b). The high biocompatibility of IONPs is due to well-controlled cell homeostasis by uptake, excretion and storage (Chenga et al. 2005).

However, nickel and cobalt are susceptible to oxidation and toxic, even though they exhibit a high magnetic moment, because they are not essential elements to the body like iron and thus accumulate in the body and cause illness. It is worth noting that SPIONs may induce dermal toxicity via their ability to be internalized and thereby initiate oxidative stress leading to inflammation (Murray et al. 2013).

IONPs become superparamagnetic at room temperature when their radius is below about 15 nm (Kolhatkar et al. 2013), and aggregation is a common phenomenon among SPIONs (Wu et al. 2015). Therefore, bare SPIONs are coated against aggregation by either non-magnetic or magnetic shell (Zenga et al. 2004). Usually, the type of coatings has an impact on the heating efficiency of the core through modifying the surface properties. Details on the types of shells used to protect IONPs and their effect over magnetic properties will be discussed.

Among iron oxides, magnetite (${\text{Fe}}_{3} {\text{O}}_{4}$) and maghemite (γ-${\text{Fe}}_{2} {\text{O}}_{3}$) are very popular candidates and have unique magnetic properties suitable for biomedical applications.

Iron metal (Fe) has a higher magnetization than magnetite and maghemite. However, Fe is highly susceptible to oxidation, which limits its use for biomedical applications. Qiang et al. synthesize oxidative stable Fe-core MNPs coated with iron oxide and having an increasing Ms from about 80 emu/g (at the cluster size of 3 nm) to 200 emu/g (at the size of 100 nm) (Qiang et al. 2006).

In general, MNPs are coated with a selected material to enhance their colloidal stability and biocompatibility or to offer them the capacity to functionalize the surface, like in the case of a coating of silica (Si${\text{O}}_{2}$) (Rittikulsittichai et al. 2013). Furthermore, coating can be used to modify MNPs surface to increase their Ms and consequently increase the SAR.

Studies show that coating MNPs with non-magnetic material, for example ${\text{Fe}}_{3} {\text{O}}_{4}$ coated with Si${\text{O}}_{2}$ (Larumbe et al. 2012a, b), will reduce Ms (from 72 emu/g to 37 emu/g) and hence SAR (from 1.5 ± 0.1 to 1.08 ± 0.04 W/g) as compared to uncoated MNPs. The decrease in Ms was attributed to the enhanced surface spin effects, and thus not all the IONPs mass contribute to Ms. Furthermore, the effective anisotropy constant ‘$K_{\text{eff}}$’ increases due to the strain and surface spin disorders created by Si${\text{O}}_{2}$ coating, and the blocking temperature $T_{B}$ experiences similar variations since $T_{B}$ is defined as the product of the $K_{\text{eff}}$ and the volume of the nanoparticles ‘V’ (Eq. 15) (Coşkun et al. 2010).

15 $$ T_{B} = \frac{{K_{\text{eff}} \cdot V}}{{25 \cdot k_{B} }} $$

Surface spin effect (or surface spin disorder) is the result of the surface electrons engagement in the bond with the coating material, which no longer participate in the magnetic super-exchange bonds between metal cations (example: Fe–O–Fe), and thus reduce the coordination between surface spins (Kodama et al. 1996).

${\text{Fe}}_{3} {\text{O}}_{4}$ NPs coated with Si${\text{O}}_{2}$ and functionalized with propylamine groups showed higher magnetization saturation (Ms ≈ 42 emu/g) than uncoated ${\text{Fe}}_{3} {\text{O}}_{4}$ (Ms ≈ 27 emu/g), where both were synthesized by thermal decomposition in oleic acid (Woo et al. 2005). It seems that the surface of ${\text{Fe}}_{3} {\text{O}}_{4}$ is magnetically more active in ${\text{Fe}}_{3} {\text{O}}_{4}$ NPs coated with silica-propylamine than that of uncoated ${\text{Fe}}_{3} {\text{O}}_{4}$ covered with oleic acid.

On the contrary, ${\text{Fe}}_{3} {\text{O}}_{4}$ NPs coated with silica-propylamine showed slightly lower magnetization saturation (Ms ≈ 58 emu/g) than uncoated ${\text{Fe}}_{3} {\text{O}}_{4}$ (Ms ≈ 60 emu/g) (Yamaura et al. 2004), where ${\text{Fe}}_{3} {\text{O}}_{4}$ NPs were obtained by co-precipitation in aqueous medium. The contradictory results of these two studies suggest that the synthesis and coating methods can be tailored to enhance the magnetic properties of the MNPs.

Capping Co-MNPs with metallic shell (such as Cu or Au) provides us a high tuning opportunity over the magnetic properties (for example, enhanced surface anisotropy and higher blocking temperature), due to the bonding of the d-orbital electrons of the core to the conduction band orbitals of the capping layer (Luis et al. 2006). This suggests that the surface anisotropy is mainly determined by the electronic states of the core–shell metals and, therefore, it could be tuned by choosing materials with appropriate electronic band structures.

For hyperthermia applications, an SLP of 1000 W/g is necessary at 100 kHz and 20 mT (human-compatible conditions). By taking advantage of the exchange coupling between a magnetically hard core (Co${\text{Fe}}_{2} {\text{O}}_{4}$) and soft shell (Mn ${\text{Fe}}_{2} {\text{O}}_{4}$), MNPs exhibiting a significant enhancement in SLP have been developed (Lee et al. 2011). Various combinations of core–shell nanoparticles tuned Ms of the single-component MNPs to achieve high SLP while maintaining the superparamagnetism. For example ${\text{Zn}}_{0.4} {\text{Co}}_{0.6} {\text{Fe}}_{2} {\text{O}}_{4}$ core and ${\text{Zn}}_{0.4} {\text{Mn}}_{0.6} {\text{Fe}}_{2} {\text{O}}_{4}$ shell MNPs have an SLP of 3866 W/g and thus exhibit 1.7 times higher SLP than that for ${\text{CoFe}}_{2} {\text{O}}_{4}$(core) ${\text{MnFe}}_{2} {\text{O}}_{4}$(shell) MNPs (2274.12 W/g) and 34 times larger than that for commercial Feridex ${\text{Fe}}_{3} {\text{O}}_{4}$ NPs (114 W/g).

Spherical Mn ${\text{Fe}}_{2} {\text{O}}_{4}$ SPMNPs show lower SLP of 411 W/g (*r* = 15 nm) when compared to that of Mn ${\text{Fe}}_{2} {\text{O}}_{4}$(core) Co ${\text{Fe}}_{2} {\text{O}}_{4}$(shell) (*r* = 15 nm) where SLP is about 3034 W/g (Noh et al. 2012). Clearly, core–shell design has the advantage in achieving large SLP while keeping the superparamagnetism of the nanoparticle. In the same work, cubes of Co ${\text{Fe}}_{2} {\text{O}}_{4}$ coated with ${\text{Zn}}_{0.4} {\text{Fe}}_{2.6} {\text{O}}_{4}$ showed a 4-fold increase in coercivity as compared to the core alone. This increase is consequently followed by a dramatically higher SAR for the shell-core MNPs (10,600 W/g) when compared to that of MNPs composed of just the core (4060 W/g).

Many efforts have been dedicated toward understanding the relationship between the shape of MNPs and their magnetic properties. Several studies showed that the Ms is proportional to the volume of the particle (*V*) with the same crystalline composition but different shape (Chou et al. 2009; Shevchenko et al. 2003), due to the decrease of the surface-to-volume ratio and consequently surface spin disorder. For example, considering MNPs having the same unit size (*d*) (where ‘*d*’ corresponds to the side length for nanocubes, the width for nanorods and the diameter for nanospheres), the *V* of nanocube is higher than the *V* of nanorod, and the *V* of nanosphere is lower than the *V* of nanorod. Therefore, the same order of Ms is expected (Ms of nanocube > Ms of nanorod > Ms of nanosphere).

A study on the effect of the shape of ${\text{Fe}}_{3} {\text{O}}_{4}$ NPs over its saturation magnetization is done by Zhen et al. (Zhen et al. 2011). The authors observed a higher Ms for the cubic shape (Ms = 40 emu/g) compared to the spherical shape (Ms = 31 emu/g), where the volume of the cube is slightly higher than that of the sphere ($V_{\text{cube}}$ > $V_{\text{sphere}}$). They attributed the lower magnetization of spherical ${\text{Fe}}_{3} {\text{O}}_{4}$ NPs to their crystalline defect structure or greater degree of oxidation and non-magnetic iron oxide (${\text{Fe}}_{2} {\text{O}}_{3}$) content.

According to Noh et al. (2012), the cubic shape of ${\text{Zn}}_{0.4} {\text{Fe}}_{2.6} {\text{O}}_{4}$ has a higher Ms (165 emu/g) value than the spherical shape (145 emu/g) with the same volume. In fact, the surface of the cube shape has a smaller surface anisotropy since its topology comprises low energy facets. As a result, disordered magnetic spins in cubic NPs (4 %) are lower than in spherical NPs (8 %).

However, in a study done by Montferrand et al. on ${\text{Fe}}_{3} {\text{O}}_{4}$ NPs (Montferrand et al. 2013) Ms for the cubic shape (40 emu/g) is lower than the spherical shape (80 emu/g) of the same size. Unexpected Ms could be related to size polydispersity and polymorphism detected in TEM images.

Magnetic properties are also defined by the atomic state of the elements, especially the number of unpaired valence electrons. For example, Fe(III) have five unpaired electrons and thus a moment of 5 × 1.73 = 8.65 Bohr magnetons. Moreover, the distribution of ions in the structure is another parameter responsible for the determination of the moment. For example, in an inverse spinel structure of ferrites, the magnetic moments of the cations in the octahedral sites are aligned parallel to the magnetic field, and the ones in the tetrahedral sites are antiparallel, leading to a decrease in the net moment (Lee et al. 2006).

Hence, doping MNPs with cations is of great interest in nanomedicine because it tailors the physical and magnetic properties, without affecting its crystal structure, due to the nature of the cation and its relative distribution in the tetrahedral and octahedral sites (Fantechi et al. 2012).

Lee et al. (2006) compared the crystal structure of four spinel ferrites (*M* $Fe_{2} O_{4}$): Mn${\text{Fe}}_{2} {\text{O}}_{4}$ (110 emu/g), Fe ${\text{Fe}}_{2} {\text{O}}_{4}$ (101 emu/g), Co${\text{Fe}}_{2} {\text{O}}_{4}$ (99 emu/g), and Ni${\text{Fe}}_{2} {\text{O}}_{4}$ MNPs (85 emu/g). Mn${\text{Fe}}_{2} {\text{O}}_{4}$ had a mixed spinel structure, where ${\text{Mn}}^{2 + }$ and ${\text{Fe}}^{3 + }$ occupied both octahedral and tetrahedral sites, and an inverse spinel structure where ${\text{Mn}}^{2 + }$ and ${\text{Fe}}^{3 + }$ occupied octahedral sites and only ${\text{Fe}}^{3 + }$ occupied the tetrahedral sites.

The inclusion of $Ni^{2 + }$ in the ferrite spinel structure (${\text{Ni}}_{\text{x}} {\text{Fe}}_{{3 - {\text{x}}}} {\text{O}}_{4}$ with *x* = 0, 0.04, 0.06 and 0.11) has no substantial change in the value of Ms, where ${\text{Ni}}^{2 + }$ occupy ${\text{Fe}}^{2 + }$ octahedral sites (Larumbe et al. 2012a, b). Gabal et al. examined the ${\text{Zn}}^{2 + }$ doped nickel ferrite (${\text{Ni}}_{1 - x} {\text{Zn}}_{x} {\text{Fe}}_{2} {\text{O}}_{4}$; 0 < *x* < 1) and noticed that the Ms increases by increasing Zn doping levels up to 0.5 (Jalalya et al. 2010). This behavior can be explained by the fact that magnetite (${\text{Fe}}_{3} {\text{O}}_{4}$), with a spinel structure, has ${\text{Fe}}^{3 + }$ ions occupying tetrahedral (inverse) sites and ${\text{Fe}}^{2 + }$ with ${\text{Fe}}^{3 + }$ ions residing in the octahedral sites. During cation exchange ${\text{Fe}}^{2 + }$ in octahedral site is replaced by ${\text{Ni}}^{2 + }$ and ${\text{NiFe}}_{2} {\text{O}}_{4}$ is formed. Since the tetrahedral and octahedral sites are antiferromagnetically coupled, the net moment of Ni ferrite equals the moment of octahedral site (${\text{Ni}}^{2 + }$, ${\text{Fe}}^{3 + }$) minus the moment of tetrahedral (${\text{Fe}}^{3 + }$). The inclusion of non-magnetic ${\text{Zn}}^{2 + }$ in ${\text{NiFe}}_{2} {\text{O}}_{4}$ substitutes ${\text{Ni}}^{2 + }$ then occupies a tetrahedral site and force magnetic ${\text{Fe}}^{3 + }$ to migrate to octahedral site and, as x increases. As a result, the net moment increases due to the decrease in fraction of moment of tetrahedral site and an increase in the moment of octahedral sites (Jalalya et al. 2010).

FeCo MNPs usually exhibit high Ms values (122–230 emu/g) compared with Co${\text{Fe}}_{2} {\text{O}}_{4}$ MNPs (Chaubey et al. 2007), due to the absence of the non-magnetic oxygen component (Zhang et al. 2012). However, the ease of oxidation in the presence of air is the key issue for these alloys (Zhang et al. 2012).

**Fig. 5:**
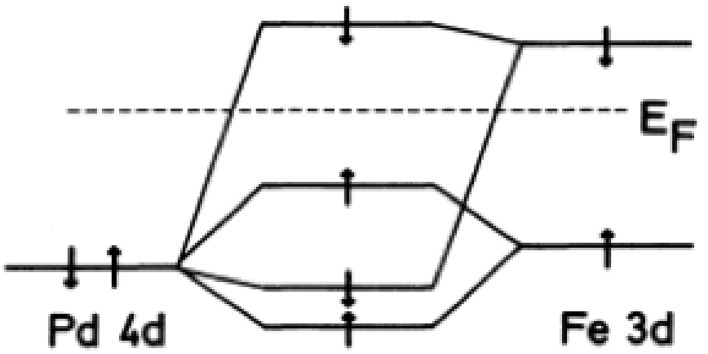
Illustration of the covalent interaction between Fe 3d and Pd 4d orbitals (reproduced from Van Acker et al. 1991)

Palladium metal is a non-magnetic element, but tends to order ferromagnetically when alloyed with a small amount of magnetic transition metal impurities (such as Fe, Co and Ni 3d metals) (Crangle and Scott 1965). A polarization of Pd atom by a magnetic impurity is due to the hybridization and exchange between 4d and 3d orbitals (Fig. 5) (Van Acker et al. 1991).

The appearance of ferromagnetism can be explained by the large density of states at the Fermi level ($E_{F}$).

Paulus and Tucker (1995) proposed for the first time PdCo seeds for thermal treatment of tumors. PdCo thermoseeds (typically rod shape where *d* = 1 mm and *L* = 1–2 cm) are permanently implanted into the cancerous tissue, and thus the patient can be scheduled for activation of the thermoseeds at intervals of minimal thermotolerance (Paulus and Tucker 1995). The authors developed a new approach to treat prostate cancer, post-radiotherapy, using these thermoseeds. During thermotherapy, PdCo rods heat up when exposed to an alternative magnetic field (due to eddy current) to a specific temperature (Curie temperature), at which the alloy goes from being magnetic to non-magnetic, and ceases to heat up and it simply maintains the Curie temperature as long as it remains in the magnetic field (Paulus and Tucker 1995).

Deger et al. (2002) conducted a clinical study on the treatment of patients with localized prostate cancer with a magnetic hyperthermia, using self-regulating PdCo thermoseeds, after radiotherapy. During hyperthermia, PdCo thermoseeds heating temperatures were between 42 and 46 °C with a Curie temperature of 55 °C. The initial median prostate-specific antigen (PSA) value was 11.6 ng/ml, and then decreased to 1.3 and 0.55 ng/ml after 12 and 24 months, respectively, after the therapy. Moreover, PdCo seeds proved to be biocompatible and do not show major complication during the treatment, and remain in the prostate during follow up (Deger et al. 2002).

According to Brezovich and Meredith (1989), a heat production rate of 200 mW/cm is adequate for most clinical application. El-Sayed et al. calculate the power dissipated from ${\text{Pd}}_{89.2} {\text{Co}}_{10.8}$,${\text{Pd}}_{73} {\text{Ni}}_{27}$ and ${\text{Cu}}_{29.6} {\text{Ni}}_{70.4}$ ferromagnetic seeds, having a rod shape with a diameter of 0.9 mm diameter and a 5.5 cm length as function of temperature (El-Sayed et al. 2007). At 20 °C, the heating power of ${\text{Pd}}_{89.2} {\text{Co}}_{10.8}$ was about 171 mW/g, and 150 mW/g for ${\text{Pd}}_{73} {\text{Ni}}_{27}$. The ${\text{Cu}}_{29.6} {\text{Ni}}_{70.4}$ seed showed a much smaller heating power of 80 mW/g. Therefore, ${\text{Pd}}_{89.2} {\text{Co}}_{10.8}$ seed exhibited the highest heating power to treat localized tumors compared with the other two alloys.

Iron based-MNPs have been widely studied for nanomedicine (especially for cancer treatment) and palladium-cobalt alloys have not received significant attention. Although Pd and Co are toxic elements, PdCo alloy has a higher stability and resistance to corrosion (Wataha et al. 1991) compared to Fe-based alloy (Arbab et al. 2005). Moreover, the researches done over PdCo thermoseeds are very promising and encouraging to develop new MNPs candidates for thermotherapy made of PdCo alloys.

## Nanotoxicity of biomaterials

Considering the wide preclinical and clinical applications of magnetic iron oxide NPs in nanomedicine, it is crucial to understand the potential nanotoxicity associated with exposure to these NPs and especially the physiological effects produced by the surface coatings used for functionality.

The work of Pisanic et al. (2007) showed that the intracellular delivery of 0.15–15 mm of iron oxide (${\text{Fe}}_{2} {\text{O}}_{3}$) NPs may adversely affect cell function and results in a dose-dependent diminishing viability and capacity of PC12 cells (rat pheochromocytoma cell line) to differentiate, in response to nerve growth factor.

In fact, uncoated iron oxide NPs have a low solubility that can lead to their precipitation and a high rate of agglomeration under physiological conditions (Lei et al. 2013). Coating these NPs aims to stabilize their surfaces against agglomeration and dissolution, and allows the grafting of biomolecules (such as antibodies and drugs) (Sadeghiani et al. 2005). However, the type of surface-coating materials is important to determine the toxicity of coated NPs.

The cytotoxic potential of iron oxide NPs with a range of surface coatings has been extensively investigated. Hilger et al. (2003) estimated the cytotoxic potential of cationic/anionic coated magnetite (${\text{Fe}}_{3} {\text{O}}_{4}$) nanoparticles by measuring the succinate dehydrogenase activity in human adenocarcinoma cells (BT-20). Cationic particles showed to induce the strongest decrease in cell survival rates of BT-20 cells (0 ± 0 after incubation for 72 h) for a concentration of 20 ng/cell. This is due to some strong electrostatic bindings to cellular membranes. On the other hand, Berry et al. (2003) found that dextran-coated iron oxide NPs could induce cell death and reduce proliferation of human fibroblasts during internalization. Significant membrane disruptions were observed in fibroblasts cells, including possible apoptosis and aberrations in cell morphology, causing decreases in cells motility (Berry et al. 2004).

Recent studies show that ${\text{Fe}}_{3} {\text{O}}_{4}$ NPs can affect the cellular functionality by altering the level of transferrin receptor expression and can change the cellular proliferation capacity by altering the expression of cyclins and cyclin-dependent kinases in cell cycle (Schäfer et al. 2007; Huang et al. 2009). Moreover, researchers are finding evidence that ${\text{Fe}}_{3} {\text{O}}_{4}$ NPs exposure can produce mutagenic effects including: chromosomal aberrations, DNA strand breakage, oxidative DNA damage and mutations (Koedrith et al. 2014). Other research has reported that the excess of iron exposure has been found to cause elevated ROS generation through the Fenton reaction, resulting in oxidative stress that damages DNA, lipids and proteins, consequently resulting in carcinogenesis (Toyokuni 1996). These findings confirm previous reports that the presence of intracellular ${\text{Fe}}_{3} {\text{O}}_{4}$ nanoparticle constructs can result in significant changes in cell behavior and viability (Buyukhatipoglu and Clyne 2011).

Upon administration into tumor tissue, MNPs interact with blood components, where thousands of biomolecules compete for limited space on an NP surface (Cedervall et al. 2007), due to van der Waal’s interactions, electrostatic interactions, hydrogen bonding and/or hydrophobic interactions (Hlady and Buijs 1996). As a result, MNPs acquire a dynamic exchange plasma proteins layer, so-called ‘corona’ (Cedervall et al. 2007), in which competitive displacement of earlier adsorbed proteins by other proteins with stronger binding affinities takes place and is referred to as ‘Vroman Effect’ (Hirsh et al. 2013). Thus, the identity, organization and residence time of these proteins determine the way cells interact with NPs (Cedervall et al. 2007). Moreover, the adsorbed proteins identity and their total amount showed to be strongly dependent on the particle surface chemistry (like surface composition, charge, topography and area) (Hlady and Buijs 1996).

Studies show that plasma proteins, including immunoglobulins and complement proteins, once adsorbed to NPs surfaces it target the particles as pathogens for clearance (called ‘opsonization’) by the reticulo-endothelial system and mononuclear phagocytic system (Ehrenberg et al. 2009). In fact, the immune system may recognize the proteins as native or as foreign pathogen depending on whether the proteins bind or not to immune cells receptors. Following proteins adsorption, platelets cells adhesion and activation on NPs may occur via interaction of adhesion receptors with the adsorbed blood proteins such as fibrinogen, fibronectin, vitronectin, and the von Willebrand factor (Nygren et al. 1995; Elam and Nygren 1992). As a result, inflammatory cells (primary polymorphonuclear leukocytes) migrate from the blood toward the NPs, triggered by chemoattractants released from activated cells (Franz et al. 2011). Inflammatory cells’ adsorption over the protein-coated NPs surface, due to protein ligands of integrins, leads to an acute or chronic inflammation (Nimeri et al. 2002).

The concept of inert biomaterials points out the need of strategies for improving implant integration, to avoid foreign body reactions. It was shown that when macrophages are cultured on surface-modified polymers displaying hydrophobic, hydrophilic and/or ionic chemistries, they change their protein expression profiles and cytokine/chemokine responses (Dinnes et al. 2007). Consequently, current studies in the design of such biomaterials include passive modulation of the surface chemistry, to limit immune responses. For example, polyethylene glycol (PEG)-modified surface reduces protein adsorption due to its sterically hindered and hydrophilic coating (Torchilin and Papisov 1994), and this leads to more blood circulation of PEG-coated NPs. On the other side, functionalization of the surface with bioactive molecule such as adhesion sites (Kao and Lee 2001), anti-inflammatory drugs (Franchimont et al. 2002) and growth factors (Barrientos et al. 2008) is also a very interesting strategy for modulating or suppressing inflammatory responses.

MNPs can induce toxicity, not only by activating cells in a direct way as discussed above, but also indirectly by excessive tissue accumulation of free metal ions (Weir et al. 1984). It was shown that reactive oxygen species (ROS) are generated by the cells as a result of leached ions after exposure to an acidic environment, such as lysosomes (pH 4.5) (Albrecht et al. 2004). In general, most cells can tolerate a certain amount of ROS, whereas higher levels of ROS persist over a longer time and may result in cell damage and subsequent induction of toxic effects (Wang et al. 2007). Since the toxicity of the NPs is affected by the level of induced ROS, the surface must be stable against degradation to limit the quantity of free metal ions.

**Fig. 6:**
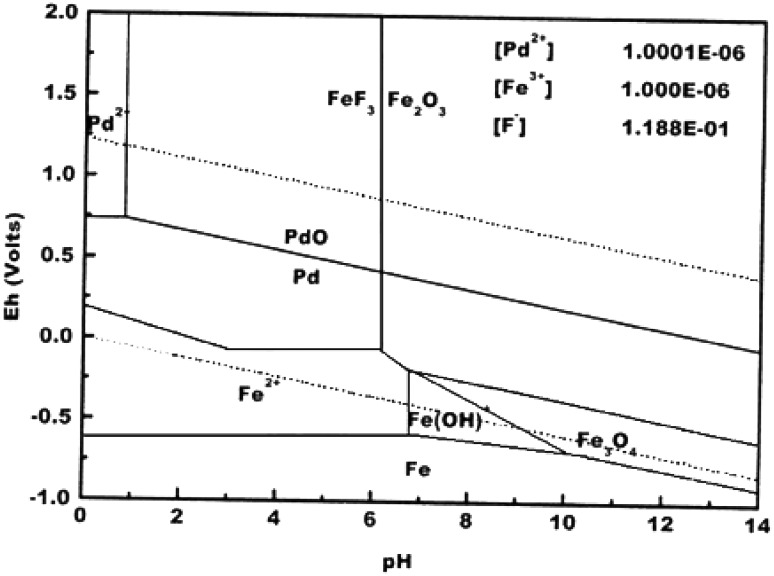
Pourbaix diagram showing iron and palladium species and water stability region (reproduced from Villicaña et al. 2007)

Potential (Eh)-pH diagram or Pourbaix diagram is essential to investigate the thermodynamic of material corrosion, by monitoring the regions of potential and pH where the metal is: unreacted (region of immunity), protected by a surface film of an oxide or a hydroxide (region of passivity) or dissolved (region of corrosion) (McCafferty 2010). Figure 6 shows the Pourbaix diagram for both iron and palladium elements in water containing fluoride ions (Villicaña et al. 2007). According to the diagram, iron will corrode and produce Fe(II) and/or Fe(III) at potential zero and at pH below 6, whereas palladium remains unreacted under these conditions. This difference in stability is due to the higher reactivity of iron towards oxidation (${\text{E}}_{{{\text{Fe}}({\text{II}})/{\text{Fe}}}}^{^\circ }$ = −0.44 V; ${\text{E}}_{{{\text{Fe}}({\text{III}})/{\text{Fe}}}}^{^\circ }$ = −0.04 V), compared with palladium (${\text{E}}_{{{\text{Pd}}({\text{II}})/{\text{Pd}}}}^{^\circ }$ = +0.915 V). Moreover, iron forms a porous oxide layer when exposed to water or air (Hill and Holman 2000), and consequently anodic (iron)/cathodic (iron oxide) sites created at the surface trigger the process of corrosion.

The reactivity of iron towards oxidation reveals the toxicity of uncoated IONPs (Pisanic et al. 2007), and suggests more study into the biocompatibility of the coatings on the long term (Hilger et al. 2003). The most important source of toxicity of IONPs is described by ‘Fenton’ and ‘Fenton like’ reactions (Eqs. (16) and (17a, 17b), respectively) (Salgado et al. 2013), in which the Fe(II) or Fe(III) reacts with ${\text{H}}_{2} {\text{O}}_{2}$ to produce ROS species.

16 $$ \begin{aligned} {\text{Fe}}\left( {\text{II}} \right) + {\text{H}}_{2} {\text{O}}_{2} \to {\text{Fe(III)}} + {\text{HO}}^{*} + {\text{HO}}^{ - } \hfill \\ k = 7 6\,{\text{M}}^{ - 1} {\text{s}}^{ - 1} \hfill \\ \end{aligned} $$

17a $$ {\text{Fe}}\left( {\text{III}} \right) + {\text{H}}_{2} {\text{O}}_{2} \to {\text{Fe(II)}} + {\text{HO}}_{2}^{*} + {\text{H}}^{ + } $$

17b $$ \begin{aligned} {\text{Fe}}\left( {\text{III}} \right) + {\text{HO}}_{2}^{*} \to {\text{Fe(II)}} + {\text{O}}_{2} + {\text{H}}^{ + } \hfill \\ k = 0.0 1 {\text{ M}}^{ - 1} {\text{s}}^{ - 1} \hfill \\ \end{aligned} $$

Free ROS species exhibit a lack of specificity with which they react (Pryor 1976), and this makes the study of the oxidative mechanism in the toxicity of iron ions very complex (Stohs and Bagchi 1995). However, ROS interactions with biological components have been classified into three types of reactions: electron transfer, radical addition and atom abstraction, and identified to cause cell damage (Moslen and Smith 1992).

The toxicity of Pd, Co pure metal and ${\text{Pd}}_{43} {\text{Co}}_{57}$ alloy was tested in vitro for dental casting (Kawata et al. 1981). It was shown that the cells multiply in the presence of Pd as much as those for the control, and keep their natural form. Whereas in the presence of Co, the cells degenerate with time and approaches zero at 72 h of incubation due to the cytoplasmic shrinkage and blister formation. On the other hand, the binary alloy ${\text{Pd}}_{43} {\text{Co}}_{57}$ enhances the cells growth and morphology compared with pure Co, showing a monotonically increase of cell multiplication like the control. These results indicate that the toxicity of Co may be avoided when alloyed with Pd.

The corrosion of the binary alloy ${\text{Pd}}_{80,8} {\text{Co}}_{19,2}$ in synthetic saliva (Goehlich and Marek 1990) produces a selective dissolution of the less noble components ‘Co’ on the surface of the alloy, leaving a Pd-enriched layer on the surface. The results of corrosion are in accordance with that of toxicity, the safety of a biomaterial largely dependent on its corrosion resistance. Therefore, pure palladium is non-toxic due to the low dissolution rate of palladium ions (Wataha and Hanks 1996), while pure Co is not stable and thus releases toxic cobalt ions.

Despite belonging to essential trace elements of the human body, the accumulation of cobalt ions is genotoxic and may cause induce necrosis with inflammatory response (Donaldsaon and Beyersmann 2005). The oral median lethal dose (${\text{LD}}_{50}$) for soluble Co salts has been estimated to be between 150 and 500 mg/kg body weight (Donaldsaon and Beyersmann 2005). Further, very low doses of Pd are sufficient to cause allergic reactions in susceptible individuals (Kielhorn et al. 2002). Oral ${\text{LD}}_{50}$ of palladium oxide is about 4.9 g/kg body weight (Nordberg et al. 2014). Also high concentrations of Pd ions are capable of eliciting a series of cytotoxic effects (Kielhorn et al. 2002).

Electrochemical corrosion test and immersion test were performed at 37 °C for ${\text{Pd}}_{93.85} {\text{Co}}_{6.15}$ alloy sample (with a density of 11.4 g/${\text{cm}}^{3}$) in mammalian Ringer’s solution (Paulus et al. 1997). The tests results showed a long-term corrosion rate of 7.7 × $10^{ - 8}$ μm/year, and a release of 0.7 μg/l of Pd(II) with 1.8 μg/l of Co(II) per year, indicating a significantly high corrosion resistance of PdCo compared with standard surgical implants (0.04 μm/year) (Paulus et al. 1997).

**Fig. 7:**
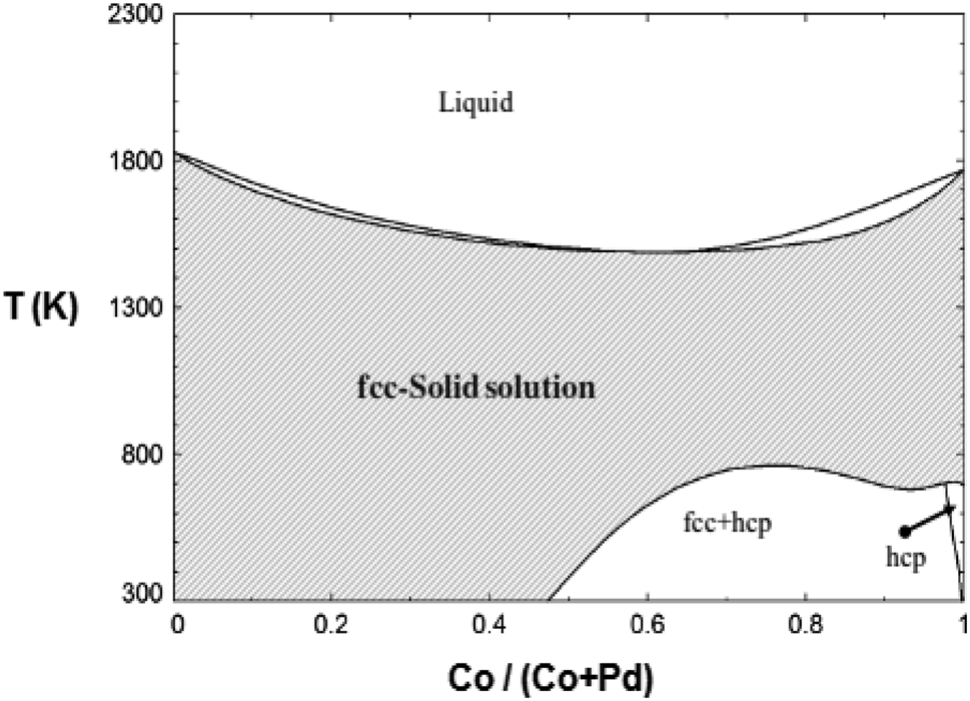
Phase diagram of PdCo system obtained from FactSage software (Bale et al. 2002)

According to the phase diagram of PdCo alloy (Fig. 7), a single phase solid solution of substitutional Co atoms in a Pd lattice is formed when the atomic percentage of Pd is higher than 53 %. Consequently, the corrosion behavior of the PdCo alloy will be similar to that of pure Pd. In fact, palladium remains unreacted at normal pH or even acidic environment, as stated in Pourbaix diagram (Fig. 6). Pure palladium corrodes only in extremely acidic medium, which is unlikely to occur in biological media. The selective dissolution of Co near or at the surface on the long-term is possible (Paulus et al. 1997), and as a result Co-depleted layer is formed. The alloy is then likely to exhibit passivation behavior of pure palladium. An additional dissolution of cobalt may occur by volume diffusion of these less noble atoms to the surface (Pickering and Wagner 1967).

Alloying Pd and Co not only induces ferromagnetism in Pd atoms but also enhances the corrosion resistance of Co atoms, which makes this alloy a good candidate for biomedical application.

Very recently using magnetic nanoparticles for enhancing the effectiveness of ultrasonic was shown (Józefczak et al. 2016). It was indicated that the effectiveness of ultrasound (US) for medical applications can be significantly improved by using sonosensitizers like magnetic nanoparticles with mean sizes of 10–300 nm. These NPs can be more effectively heated because of additional attenuation and scattering of US. Accordingly, this can enhance the thermal effect of ultrasound (US) on the tissue by increasing US absorption. The other interesting aspect using magnetic NPs is that they are able to produce heat in the alternating magnetic field (magnetic hyperthermia). This is particularly important because it introduces synergetic application of ultrasonic and magnetic hyperthermia which can lead to a promising treatment modality. In particular, it was found that in the samples with magnetic nanoparticles, the synergetic action of ultrasounds and magnetic field allowed achieving better heating effect in comparison to the heating by either US or alternating magnetic field (AMF) alone. This synergistic effect was confirmed by specific absorption rate (SAR) values. The following SAR values were, respectively, obtained: 66 mW/g for magnetic hyperthermia, 175 mW/g for ultrasonic hyperthermia, and 375 mW/g for both methods applied simultaneously. This opens the ways to future potential investigations of better utilization of NPs and ultrasound for stand-alone magnetic hyperthermia therapy applications.

## Conclusions

Magnetic NPs are frequently employed in biomedical research as drug delivery systems and/or magnetic resonance contrast agents. Nevertheless, the safety issues of these particles have not been completely solved because it is difficult to compare the cytotoxicity data since the toxic effects of NPs are influenced by many parameters (such as size distribution, surface coating, magnetic properties, etc.) (Auffan et al. 2006). Also, numerous studies showed contradicting findings since different cell types will interact with the same particle in different ways (Barua and Rege 2009). Moreover, the lack of coherence between various research activities for establishing priorities among the research needs is one reason why a toxicological profile of these particles has not yet been well documented in the literature. Therefore, along with the expanding applications of NPs and the growing numbers of consumer products containing NPs, the release of these substances into the environment is expected, and the impact of these materials is increasing significantly (Zhu et al. 2012).

In this study, we have reviewed the basics of magnetic properties and nanotoxicity of NPs for magnetic hyperthermia. Also, recent advances on the most used MNPs for biomedical application were discussed. From this study, it can be seen that despite its corrosion problem, iron oxide NPs have received considerable attention. However, new candidates such as PdCo NPs may have a great potential for magnetic hyperthermia due to their high corrosion resistance and good ferromagnetic behavior.

Some challenges need to be addressed on the design of novel NPs, which must meet the demands of a particular application. The elaboration of methods must be also significantly improved to assess the toxicity of NPs, such as reference biomaterials for safety testing. Synergetic approaches combining magnetic and ultrasounds properties must be also more investigated to improve the applicability of magnetic NPs for magnetic hyperthermia therapy.
